# Incorporating topological information for predicting robust cancer subnetwork markers in human protein-protein interaction network

**DOI:** 10.1186/s12859-016-1224-1

**Published:** 2016-10-06

**Authors:** Navadon Khunlertgit, Byung-Jun Yoon

**Affiliations:** Department of Electrical and Computer Engineering, Texas A&M University, College Station, 77843-3128 TX USA

**Keywords:** Cancer classification, Subnetwork marker identification, Protein-protein interaction network, Message passing algorithm, Topological information

## Abstract

**Background:**

Discovering robust markers for cancer prognosis based on gene expression data is an important yet challenging problem in translational bioinformatics. By integrating additional information in biological pathways or a protein-protein interaction (PPI) network, we can find better biomarkers that lead to more accurate and reproducible prognostic predictions. In fact, recent studies have shown that, “modular markers,” that integrate multiple genes with potential interactions can improve disease classification and also provide better understanding of the disease mechanisms.

**Results:**

In this work, we propose a novel algorithm for finding robust and effective subnetwork markers that can accurately predict cancer prognosis. To simultaneously discover multiple synergistic subnetwork markers in a human PPI network, we build on our previous work that uses affinity propagation, an efficient clustering algorithm based on a message-passing scheme. Using affinity propagation, we identify potential subnetwork markers that consist of discriminative genes that display coherent expression patterns and whose protein products are closely located on the PPI network. Furthermore, we incorporate the topological information from the PPI network to evaluate the potential of a given set of proteins to be involved in a functional module. Primarily, we adopt widely made assumptions that densely connected subnetworks may likely be potential functional modules and that proteins that are not directly connected but interact with similar sets of other proteins may share similar functionalities.

**Conclusions:**

Incorporating topological attributes based on these assumptions can enhance the prediction of potential subnetwork markers. We evaluate the performance of the proposed subnetwork marker identification method by performing classification experiments using multiple independent breast cancer gene expression datasets and PPI networks. We show that our method leads to the discovery of robust subnetwork markers that can improve cancer classification.

**Electronic supplementary material:**

The online version of this article (doi:10.1186/s12859-016-1224-1) contains supplementary material, which is available to authorized users.

## Introduction

In this work, we focus on one of the problems in translational genomics which is the identification of biomarkers from microarray gene expression data to classify type or state of complex disease. This problem is generally challenging and practically difficult because it normally involves with: 1) Small sample size of clinical data, 2) Large number of potential markers, and 3) Heterogeneity across patient and samples.

Several studies have been working on identifying gene markers which are selected based solely on gene expression data. These markers have shown to be useful to build classifiers for disease prediction. However, there are some limitations of these gene-based markers. For example, given two large-scale-dataset studies of breast cancer metastasis [1, 2]. Both studies tried to find out what would be the gene markers to look at in order to estimate the risk of cancer metastasis. Both of them identified around 70 gene markers with 60–70 % of accuracy. However, they shared only 3 genes in common from 55 of possible genes that might share across two platforms [3]. These gene-based markers yielded low performance on cross-dataset experiments. Afterward, many studies have been proposed to improve prediction accuracy and reproducibility of the identified biomarkers.

As cancer is a complex disease which its progression involves dysregulation of multiple genetic processes, there is an alternative approach based on the assumption that genes which are known to be in common pathways [4–8] or genes whose protein products are functionally related in protein-protein interaction (PPI) networks [9–11] should be interpreted together as a single feature. This approach analyzes gene expression data at “modular” level by integrating biological information, such as known molecular pathways or PPI networks. Many studies have shown that this “integrative approach” tends to be more robust than single gene markers and may improve classification accuracy.

This approach has drawn the attention to several studies to find what might be the effective way to integrate the expression of genes that belong to the same module. Several ideas have been proposed such as using mean or median, sum, or difference of the expression levels of the gene that belong to the same modules as modular activity. PPI network has been shown to overcome the limited numbers of known pathway information. Chuang et al. [9], one of the first studies in this field, proposed a greedy search algorithm for finding discriminative subnetwork markers. Su et al. [10] proposed dynamic programming method to identify and greedily combined paths containing differentially expressed and coexpressed genes to obtain subnetwork markers for predicting breast cancer metastasis. More recently, in our previous work [11], we utilized a message-passing clustering algorithm to identify subnetwork markers with high-accuracy disease prediction. The method is capable to simultaneously predict multiple non-overlapping subnetwork markers which may lead to cover more genes with lower computational cost compared to the existing methods.

With these advantages, we adopt our previous message-passing based approach while incorporating the topological information from the PPI network to identify the potential functional modules–or subnetworks. Initially, we adopt widely made assumptions that densely connected subnetworks may likely be potential functional modules and that proteins that are not directly connected but interact with similar sets of other proteins may share similar functionalities. We employ association indices to estimate the topological information.

Association indices have been shown to be one of powerful tools for measuring similarity between genes [12]. For example, Jaccard index has been successfully used to measure neighborhood similarity for clustering and constructing Power Graph in the work of Royer et al. [13].

In this paper, we propose a novel method for incorporating PPI network topological information to enhance identification of subnetwork markers for predicting cancer prognosis. We utilize various association coefficients to estimate the topological similarity and also apply different approaches to integrate into our previous message-passing based method. We assess the identified subnetwork markers and evaluate their discriminative power and their classification performance through experiments using publicly available independent breast cancer gene expression datasets and PPI networks.

## Materials and methods

### Datasets

In this study, we obtained two independent breast cancer microarray gene expression datasets from the public domain, which we refer to as GSE2034 [2] and NKI295 [14]. GSE2034 was profiled on the Affymetrix U133a platform (GPL96) and downloaded from the Gene Expression Omnibus (GEO) database [15]. NKI295 was profiled on Agilent Hu25K platform and downloaded from the supplement information from Chang et al. [16]. We used both datasets as published by their original studies. GSE2034 contains expression profiles of 286 breast cancer patients, NKI295 contains expression profiles of 295 patients. For 108 patients in GSE2034 and 78 patients in NKI295, metastasis had been detected within 5 years of surgery. We labeled them as “metastatic”, while the remainder was labeled as “non-metastatic”.

Four publicly available human PPI networks were used in this study which we refer to as Chuang, HPRD, GASOLINE, and BioGRID. Chuang was obtained from a previous study by Chuang et al. [9]. HPRD was downloaded from the Human Protein Reference Database Release 9 [17]. GASOLINE was obtained from the work of Micale et al. [18]. It was derived from STRING database [19] considering only experimentally verified protein interactions. BioGRID was downloaded from the Biological General Repository for Interaction Datasets version 3.4.134 (Homo Sapiens) [20]. We did not combine all the PPI networks because they were compiled based on different criteria and domain of interest.

Table 1 shows the number of unique proteins and interactions for each PPI network. BioGRID contains the largest number of interactions while HPRD contains the largest number of proteins.

**Table 1:** The number of proteins and interactions for each PPI network

PPI network	Number of unique proteins	Number of interactions
Chuang	11,203	57,235
HPRD	30,047	41,327
GASOLINE	9556	53,859
BioGRID	20,364	315,507

We overlaid the gene microarray datasets with each PPI network by mapping each gene to its corresponding protein in the network. After removing the proteins that do not have corresponding genes in both gene expression datasets, we obtained an induced networks with the statistics shown in Table 2. After data integration, the numbers of proteins are quite similar to each other. BioGRID still contains the largest number of interactions while the others contain approximately the same.

**Table 2:** The number of proteins and interactions for each induced PPI network

PPI network	Number of unique proteins	Number of interactions
Chuang	5293	26,773
HPRD	4762	18,684
GASOLINE	4277	22,253
BioGRID	5697	99,426

### Affinity propagation-based subnetwork identification

We adopt the subnetwork identification procedure from our previous study [11], where we utilized a message-passing clustering algorithm, called affinity propagation, to cluster genes whose protein products interact with each other or are closely located in PPI network. The input of this clustering algorithm is the measure of similarity between genes. We originally defined the similarity of genes based entirely on the discriminative power to distinguish between the two class labels as follows:

1 $$  s_{DP}(i,k) = t_{k}+\min{\{t_{ik}-t_{i},t_{ik}-t_{k}\}}-\alpha|t_{i}-t_{k}|  $$

where *t*_*i*_, and *t*_*k*_ are *t*-test statistics score of the log-likelihood ratio (LLR) between metastatic and non-metastatic samples of genes *i*, and *k*, respectively. *t*_*ik*_ is the *t*-test score of the summation of the LLRs of genes *i*, and *k*.

The LLR, *λ*, of gene *i*, *λ*(*x*_*i*_), is based on probabilistic inference strategy proposed in [7] and it is computed by

2 $$  \lambda(x_{i})=\log \left[ f^{1}(x_{i}) / f^{2}(x_{i}) \right],  $$

where *x*_*i*_ is the expression level of the gene *i* and *f* ^*j*^(*x*_*i*_) is the conditional Gaussian probability density function of *x*_*i*_ under phenotype *j*.

The last term is the penalty term measured by the difference between discriminative power of considering genes. The parameter, *α*, is defined between [0,1] to control this term. It is shown in our previous work [11] that the size of the network decreases as *α* gets larger. It is because a larger *α* tends to cluster genes with similar discriminative power. As a result of that, it yields small subnetworks with fewer genes.

The Eq. 1 is based on original assumptions that when considering similarity between two genes, the gene itself should have high discriminative power, combining both genes as subnetwork should increase the overall discriminative power, and both genes should have similar discriminative power.

### Incorporating topological information for computing the similarity between genes

With the assumption that the proteins corresponding to the genes in the same subnetwork should have common topological attributes, we consider two following points:

- Densely connected subnetworks may likely be potential functional modules.
- Proteins that are not directly connected but interact with similar sets of other proteins may share similar functionalities.

Based on these considerations, we incorporate the topological information of proteins in the PPI network by measuring their association coefficient–or topological similarity.

We measure topological attribute using different types of association coefficients. Let *N*_*i*_ and *N*_*k*_ be the neighborhood binary vectors of protein *i* and *k*. We define the topological similarity between proteins *i* and *k*, *s*_*T*_(*i,k*), based on different similarity indexes as follows:

1. Jaccard index: We define topological similarity, $s_{T_{J}}(i,k)$, as
  3 $$  s_{T_{J}}(i,k) = \frac{|N_{i} \cap N_{k}|}{|N_{i} \cup N_{k}|}  $$
  Jaccard index is widely used to quantify the similarity
2. Kulczyński index: This measure, $s_{T_{K}}(i,k)$, represents the average proportion of the number of common neighbors to the total number of neighbors of each protein. It is given by
  4 $$  s_{T_{K}}(i,k) = \frac{1}{2}\left(\frac{|N_{i} \cap N_{k}|}{|N_{i}|}+\frac{|N_{i} \cap N_{k}|}{|N_{k}|}\right)  $$
3. Tversky index: We define topological similarity based on Tversky index, $s_{T_{T}}(i,k)$, as
  5 $$ s_{T_{T}}(i,k) = \frac{|N_{i} \cap N_{k}|}{|N_{i} \cap N_{k}|+a_{T_{T}}|N_{i} - N_{k}|+b_{T_{T}}|N_{k} - N_{i}|}  $$
  In order to indicate the direction of similarity (asymmetric similarity), we let $a_{T_{T}}=1$ and $b_{T_{T}}=0$. This asymmetric definition lets the exemplars of the identified clusters be more densely connected than other non-exemplars. We can rewrite the equation as followings
  6 $$  s_{T_{T}}(i,k) = \frac{|N_{i} \cap N_{k}|}{|N_{i}|}  $$
  Tversky index can be viewed as a general form of Tanimoto coefficient (Jaccard index) when $a_{T_{T}}=1$ and $b_{T_{T}}=1$, and Dice coefficient when $a_{T_{T}}=0.5$ and $b_{T_{T}}=0.5$.

We do not include other similarity indices whose results are in the same order (no alteration in the ranks) because they give the same output when applying affinity propagation. For example, Dice coefficient, $\frac {(2 \cdot |N_{i} \cap N_{k}|)}{|N_{i}|+|N_{k}|}$, and Jaccard index share similar results in terms of ranking. Ochiai index (or Cosine index), $\frac {|N_{i} \cap N_{k}|}{\sqrt {|N_{i}| \cdot |N_{k}|}}$, and Geometric index, $\frac {|N_{i} \cap N_{k}|^{2}}{|N_{i}| \cdot |N_{k}|}$ provide the same ranks as of Kulczyński index.

As we focus on retrieving topological information from the PPI network, we do not make use of the number of common non-neighbor proteins |¬*N*_*i*_∩¬*N*_*k*_| in this study.

Finally, we add the topological similarity, (3), (4) and (6), to the computation of similarity between genes *i* and *k*, *s*(*i,k*), in two different ways.

1. Similarity between genes *i* and *k*, *s*(*i,k*), as a product of the topological similarity *s*_*T*_(*i,k*) and the discriminative power based similarity *s*_*DP*_(*i,k*). We define as:
  7 $$  s(i,k) = s_{T}(i,k) \cdot s_{DP}(i,k)  $$
2. Similarity between genes *i* and *k*, *s*(*i,k*), as a combination of the topological similarity *s*_*T*_(*i,k*) and the discriminative power based similarity *s*_*DP*_(*i,k*). We first scale the discriminative power based similarity *s*_*DP*_(*i,k*) into the range [0,1] as same as topological similarity’s by
  8 $$ \hat{s}_{DP}(i,k) = \frac{s_{DP}(i,k)-\min(s_{DP})}{\max(s_{DP})-\min(s_{DP})}  $$
  where *s*_*DP*_ is the set of all discriminative power based similarity of all gene pairs. Then, we combine them as follows
  9 $$  s(i,k) = \beta (s_{T}(i,k)) + (1-\beta)(\hat{s}_{DP}(i,k))  $$
  where *β*=[0,1] is used to control the magnitude between each similarity. Topological similarity, *s*_*T*_(*i,k*), has more effects as *β* increases. It should be noted that *s*(*i,k*) can be viewed as the summation of topological similarity and discriminative power based similarity when *β*=0.5.

We use the same setting for preference as in [11]. The self-similarity is set to *s*(*k,k*)=*c* for all *k*, where *s*(*i,k*)≤*c* for only 1 % of all gene pairs (*g*_*i*_,*g*_*k*_) to guarantee that every gene gets equal chance to be an exemplar at the initial stage of clustering process.

### Probabilistic inference of subnetwork activity

To estimate the modular—or subnetwork—activity of identified subnetwork, we employ the probabilistic inference method proposed in [7] which is the aggregation of the LLRs of all member genes to represent the activity level of the subnetwork markers, $A(\boldsymbol {\mathcal {G}})$. It is computed by

10 $$ A(\boldsymbol{\mathcal{G}}) = \sum_{i=1}^{n} \lambda\left(x_{i} \right),   $$

where *x*_*i*_ is the expression level of the gene *g*_*i*_ in the subnetwork $\boldsymbol {\mathcal {G}}=\left \{ g_{1},g_{2},\ldots,g_{n} \right \}$. This inference method can be viewed as the aggregation of the probabilistic evidence of the expression level of genes in the subnetworks.

### Experimental set-up

We identified subnetwork markers incorporating three different strategies to measure topological similarity which we referred to as Jaccard-based, Kulczyński-based, and Tversky-based. As mentioned previously, we used two different approaches to integrate topological similarity to measure similarity between genes: 1) Product of topological and discriminative power based similarity, namely, “product-based approach”, and 2) Linear combination of topological and discriminative power based similarity, namely, “linear-combination-based approach”. In the latter approach, we used three different values of *β*(=0.25,0.5,0.75) to investigate the impact of topological similarity to the subnetwork identification. In fact, we can also setup the experiments the other way around to find the optimal the value of *β* for each data.

After computing similarity between genes and applying affinity propagation-based subnetwork identification, all output clusters were ranked based on the *t*-test statistics score of their activity level. Then we selected the top 50 clusters with high discriminative power as the potential subnetwork markers for assessing their classification performance.

We repeated these processes to both gene expression datasets and all four PPI networks.

## Results

For comparison, we also evaluated the method proposed in [9], and [11] which we refer to as the ‘greedy’ method, and the ‘AP-based’ method, respectively. We applied the greedy method with 5 % minimum required improvement which is the same setting as originally published in [9]. In the AP-based method, we set the magnitude of the penalty term, *α*, to 0.5 by reason shown in [11] that it yields high and consistent classification performance as of smaller *α* with the smaller size of identified subnetworks compared to larger *α*.

For simplicity in displaying Tables and Figures in this section, we abbreviate Jaccard-based, Kulczyński-based, and Tversky-based to *jac*, *kul*, and *tve*, respectively. The suffixes, _*p*, and _*lc* are appended to indicate product-based approach, and linear-combination-based approach, respectively.

### Statistics of the subnetwork markers

Table 3 shows the average size of top 50 highly discriminative subnetwork markers identified by each method on GSE2034 and NKI295. Each column shows the results for each PPI network. The average size of markers identified by product-based and linear-combination-based approach is similar to the original AP-based method. We can clearly see that the average size of top markers identified by the proposed method and AP-based is larger than the greedy-based.

**Table 3:** The average size of top 50 highly discriminative subnetwork markers from GSE2034 and NKI295

Gene expression dataset = GSE2034					
		Chuang	HPRD	GASOLINE	BioGRID
Greedy		3.1	3.26	3.54	3.66
AP-based		36.28	35.78	34.18	38.78
jac_p		18.06	19.94	19.58	29
kul_p		21.16	25.32	22.48	36.28
tve_p		34.48	45.26	45.98	61.8
jac_lc	*β*=0.25	18.3	21.36	23.14	34
	*β*=0.5	15.08	15.38	16.44	24.24
	*β*=0.75	13.28	16.34	13.44	19.18
kul_lc	*β*=0.25	24	30.14	28.68	39.02
	*β*=0.5	18.98	22.86	24.18	38.32
	*β*=0.75	16.06	19.12	20.84	30.98
tve_lc	*β*=0.25	34.1	46.58	43.44	53.54
	*β*=0.5	28.98	43.8	45.5	71.24
	*β*=0.75	22.92	44.78	46.32	82.66
Gene expression dataset = NKI295					
		Chuang	HPRD	GASOLINE	Biogrid
Greedy		4.12	3.68	4.46	4.42
AP-based		31.34	30.32	28.78	34.66
jac_p		14.62	16	18.94	27.72
kul_p		12.3	22.5	26.9	33.34
tve_p		28.22	42.24	49.9	57.1
jac_lc	*β*=0.25	15.14	16.8	19.66	30.06
	*β*=0.5	13.38	12.44	13.68	22.66
	*β*=0.75	11.54	12.88	10.78	17.98
kul_lc	*β*=0.25	14.8	24.6	27.06	39.26
	*β*=0.5	15.9	18.5	23.28	33.96
	*β*=0.75	13.7	17.12	17.22	27.14
tve_lc	*β*=0.25	30.76	41.78	48.66	52.44
	*β*=0.5	27.26	41.62	50.7	72.88
	*β*=0.75	18.52	43.22	48.24	81.42

As we can see from Table 3, the average size of top 50 highly discriminative subnetwork markers increases as the PPI network with larger number of interactions and unique proteins is used. This trend can be clearly seen when BioGRID is employed. Among product-based approach group, Tversky-based similarity, *tve*_*p*, yields larger subnetworks. In linear-combination-based approach, we can see that the average size decreases as *β* increases in most cases. However, we cannot see this trend distinctly in Tversky-based, *tve*_*lc*. The main reason is that Tversky-based similarity mostly provides higher similarity index compared with the others as it is designed to indicate the direction of the similarity. For instance, when a gene shares all of its neighbors with another gene (|*N*_*i*_ ∩ *N*_*k*_| = |*N*_*i*_|), it returns the maximum similarity ($s_{T_{T}}(i,k)=1$), whereas the other topological similarities yield lower because they depend on the number of neighbors the both genes.

As defined in Eq. 9, the clustering process relies more on topological information as *β* gets larger. Therefore, in this case, more genes tend to be clustered into the same subnetwork.

We can see the similar trends for the number of unique genes in top 50 discriminative subnetwork markers as shown in Table 4. We can also clearly see that the top markers identified by the proposed method and AP-based cover more genes than the greedy-based. The larger unique genes covered show that the proposed method may increase the chance to discover genes that are not known to be related to the disease. This also means the higher probability of identifying new subnetwork and pathway.

**Table 4:** The number of unique genes in top 50 highly discriminative subnetwork markers from GSE2034 and NKI295

Gene expression dataset = GSE2034					
		Chuang	HPRD	GASOLINE	Biogrid
Greedy		130	121	140	139
AP-based		1814	1789	1709	1939
jac_p		903	997	979	1450
kul_p		1058	1266	1124	1814
tve_p		1724	2263	2299	3090
jac_lc	*β*=0.25	915	1068	1157	1700
	*β*=0.5	754	769	822	1212
	*β*=0.75	664	817	672	959
kul_lc	*β*=0.25	1200	1507	1434	1951
	*β*=0.5	949	1143	1209	1916
	*β*=0.75	803	956	1042	1549
tve_lc	*β*=0.25	1705	2329	2172	2677
	*β*=0.5	1449	2190	2275	3562
	*β*=0.75	1146	2239	2316	4133
Gene expression dataset = NKI295					
		Chuang	HPRD	GASOLINE	Biogrid
Greedy		114	110	118	150
AP-based		1567	1516	1439	1733
jac_p		731	800	947	1386
kul_p		615	1125	1345	1667
tve_p		1411	2112	2495	2855
jac_lc	*β*=0.25	757	840	983	1503
	*β*=0.5	669	622	684	1133
	*β*=0.75	577	644	539	899
kul_lc	*β*=0.25	740	1230	1353	1963
	*β*=0.5	795	925	1164	1698
	*β*=0.75	685	856	861	1357
tve_lc	*β*=0.25	1538	2089	2433	2622
	*β*=0.5	1363	2081	2535	3644
	*β*=0.75	926	2161	2412	4071

Next, we studied the overlap between the top 50 highly discriminative subnetwork markers identified on different gene expression datasets. The proposed method yield larger overlap when comparing to all of the previous methods as shown in Table 5. Again, similar trends as in Table 3 can also be observed here. The larger overlaps show that more of common genes are covered and shared among identified subnetworks from independent dataset from different platforms. This may lead us to more robust classifiers, we demonstrate the robustness by providing classification performance charts showing that the experimental results from the proposed method are consistent in the next section.

**Table 5:** Overlap between the top subnetwork markers identified on different gene expression datasets

		Chuang	HPRD	GASOLINE	Biogrid
Greedy		5.63 %	4.05 %	4.88 %	3.96 %
AP-based		24.90 %	28.70 %	27.71 %	23.89 %
jac_p		37.89 %	29.28 %	32.01 %	31.97 %
kul_p		15.38 %	27.52 %	26.49 %	28.26 %
tve_p		25.80 %	44.15 %	50.57 %	42.33 %
jac_lc	*β*=0.25	39.10 %	22.54 %	26.55 %	30.20 %
	*β*=0.5	53.51 %	26.68 %	26.87 %	37.94 %
	*β*=0.75	54.55 %	31.74 %	26.67 %	40.12 %
kul_lc	*β*=0.25	12.73 %	24.47 %	27.90 %	28.50 %
	*β*=0.5	39.86 %	28.29 %	31.18 %	33.26 %
	*β*=0.75	50.61 %	35.53 %	31.42 %	40.73 %
tve_lc	*β*=0.25	27.53 %	44.47 %	46.75 %	36.57 %
	*β*=0.5	32.14 %	43.47 %	52.41 %	54.90 %
	*β*=0.75	32.99 %	50.94 %	57.71 %	69.05 %

Additionally, we analyzed enriched functions of the genes in the subnetwork markers using Panther [21], a web-based system designed to facilitate analysis of large numbers of genes and provide comprehensive function information which includes up-to-date comprehensive Gene Ontology (GO) annotations (GO database version 1.2, released 2016-05-20 with 44,588 total annotations). An example of the enrichment analysis of the top 50 highly discriminative subnetworks identified using *tve*_*p* method on GASOLINE is shown in Table 6. We can see that the genes in identified subnetworks from different gene expression datasets also share common GO terms.

**Table 6:** The number of genes in top 50 highly discriminative subnetwork markers from *tve*_*p* method on GASOLINE categorized by their GO terms

Ontology: Molecular function			
GO term	GO id	GSE2034	NKI295
transporter activity	GO:0005215	240	251
translation regulator activity	GO:0045182	37	41
protein binding transcription factor activity	GO:0000988	35	42
enzyme regulator activity	GO:0030234	193	205
catalytic activity	GO:0003824	1146	1221
channel regulator activity	GO:0016247	5	6
receptor activity	GO:0004872	346	370
nucleic acid binding transcription factor activity	GO:0001071	307	316
antioxidant activity	GO:0016209	8	6
structural molecule activity	GO:0005198	226	260
binding	GO:0005488	1237	1330
Ontology: Cellular component			
GO term	GO id	GSE2034	NKI295
synapse	GO:0045202	15	15
cell junction	GO:0030054	13	11
membrane	GO:0016020	288	290
macromolecular complex	GO:0032991	213	214
extracellular matrix	GO:0031012	50	58
cell part	GO:0044464	765	794
organelle	GO:0043226	411	441
extracellular region	GO:0005576	151	153
Ontology: Biological process			
GO term	GO id	GSE2034	NKI295
cellular component organization or biogenesis	GO:0071840	278	309
cellular process	GO:0009987	1559	1679
localization	GO:0051179	536	577
apoptotic process	GO:0006915	174	194
reproduction	GO:0000003	104	118
biological regulation	GO:0065007	886	933
response to stimulus	GO:0050896	547	593
developmental process	GO:0032502	634	692
rhythmic process	GO:0048511	3	1
multicellular organismal process	GO:0032501	393	413
locomotion	GO:0040011	20	24
biological adhesion	GO:0022610	127	147
metabolic process	GO:0008152	1773	1876
growth	GO:0040007	1	3
immune system process	GO:0002376	314	342

### Discriminative power of the subnetwork markers

**Fig. 1:**
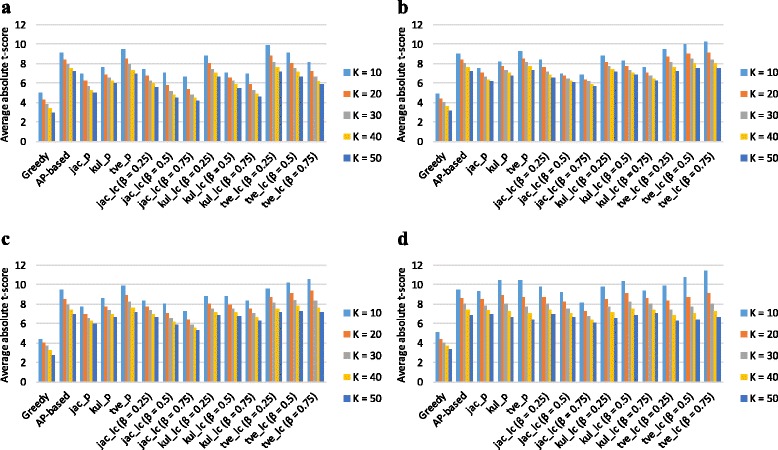
Discriminative power of subnetwork markers identified on GSE2034 by different methods. We computed the average absolute *t*-test score of the top *K*=10, 20, 30, 40, and 50 subnetwork markers identified on GSE2034 by various methods for the following PPI datasets: **a** Chuang, **b** HPRD, **c** GASOLINE, and **d** BioGRID

We evaluated the discriminative power of the subnetwork markers based on the same procedure as previously used in these studies [6–8, 10]. We computed the *t*-test score of the inferred subnetwork activity level. And then we sorted the absolute value in descending order. The average absolute *t*-test score of the top *K*=10,20,30,40,50 subnetwork markers is shown in Fig. 1. We can see that the discriminative power of subnetwork markers identified by product-based approach, and linear-combination-based approach are considerably higher than the result of the greedy method. Among product-based approach group, Tversky-based yields the highest in most of the results.

**Fig. 2:**
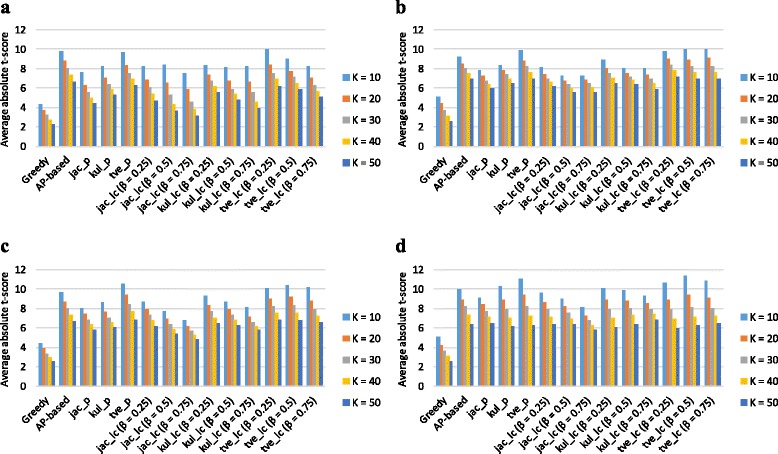
Discriminative power of subnetwork markers across independent gene expression datasets. The markers were identified and ranked on GSE2034 and their discriminative power was evaluated on NKI295. We computed the mean absolute *t*-score of the top *K*=10, 20, 30, 40, and 50 markers by different methods for the following PPI datasets: **a** Chuang, **b** HPRD, **c** GASOLINE, and **d** BioGRID

We also assessed how the subnetwork markers identified on specific gene expression dataset perform in another independent dataset. We sorted the subnetwork markers based on their *t*-test score of the inferred subnetwork activity level on one dataset and we reevaluated the discriminative power on the other dataset. As shown in Fig. 2, we can see that the trends of discriminative power of subnetwork markers across different gene expression datasets are similar to those observed in Fig. 1. The analysis of discriminative power of the subnetwork markers identified on NKI295 data also shows a similar trend (Figures S1 and S2 in Additional file 1).

About the impact of different PPI networks, the PPI network with larger number of interactions tends to yield the higher discriminative power. One of the reasons may be that it contains more topological information which may help to measure the similarity between genes. As intuitively expected, we can see that BioGRID is advantageous to the other PPI networks because it contains the largest number of interactions (as shown in Figures 1 d and Additional file 1: Figure S1(d)).

### Evaluating the reproducibility of the identified subnetwork markers

In order to evaluate the reproducibility of subnetwork markers, we performed five-fold cross-validation experiments based on a similar set-up that has been commonly used in previous studies [6–11], where the entire process was repeated for 100 random partitions.

**Fig. 3:**
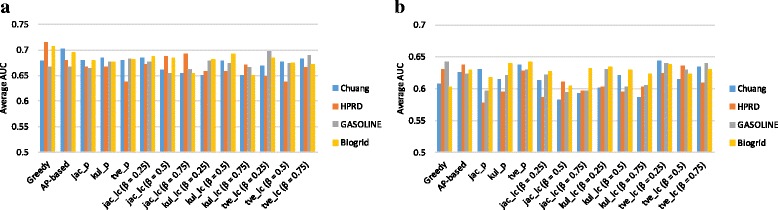
Reproducibility of subnetwork markers identified by various methods. The bars show the cross-dataset classification performance (average AUC) of different methods. **a** GSE2034 was used for identifying the potential markers and NKI295 was used for training and evaluating the classifier, **b** We repeated as NKI295 was used for identifying the markers and GSE2034 was used for training and evaluation of the classifier

We identified potential subnetwork markers and selected the top 50 subnetworks as a feature set for the classifier on one gene expression dataset. After that, we built the linear discriminant analysis (LDA) classifiers based on the selected features and evaluated the accuracy on the other dataset. The classification performance assessed by the area under ROC curve (AUC) is shown in Fig. 3. We can see that both product-based approach and linear-combination based approach yield consistently high performance across different gene expression datasets and PPI networks.

In this work, we use the term, ‘reproducibility’ in the sense of the ability to identify common discriminative genes or subnetworks across different independent datasets. Therefore, using these subnetworks as biomarkers for disease classification may lead to consistent performance. Furthermore, in terms of reproducibility in practical usage, the AP-based methods, including our proposed methods, cost less computation time compared to the greedy algorithm as shown in [11].

## Conclusion

In this paper, we propose a novel method that incorporates topological information to identify subnetwork markers that can be used in cancer prognosis prediction. We demonstrate how widely used association coefficients, such as Jaccard index, Kulczyński index, and Tversky index can be utilized to measure topological similarity. Also, we show how to integrate these measures by two different approaches, product-based, and linear-combination based.

Based on our experimental results, Tversky-based strategy is most suitable to measure similarity between genes when the direction of interaction is involved. It yields consistently high discriminative power across different datasets. Furthermore, utilizing the larger PPI network with larger number of unique proteins and interactions, such as BioGRID, may lead to the better subnetwork identification with higher classification performance.

The proposed method considerably increases the coverage of genes and also the overlap of genes when identified across different independent datasets. Through extensive evaluations using various independent breast cancer gene expression datasets and PPI networks, the experimental results show that our method leads to the identification of robust and reproducible subnetwork markers that may lead to better cancer classification.

## Additional file

Additional file 1 Supplementary materials. **Figure S1:** Discriminative power of subnetwork markers identified on NKI295 by different methods. **Figure S2:** Discriminative power of subnetwork markers across independent gene expression datasets. (PDF 1260 kb)
