# Supplementary material for: Incorporating topological information for predicting robust cancer subnetwork markers in human protein-protein interaction network
Source: BMC Bioinformatics. 2016 Oct 6;17(Suppl 13):351. doi: 10.1186/s12859-016-1224-1 (PMC5073942; doi:10.1186/s12859-016-1224-1)
Supplement: Supplementary file 1 — Supplementary materials. Figure S1: Discriminative power of subnetwork markers identified on NKI295 by different methods. Figure S2: Discriminative power of subnetwork markers across independent gene expression datasets. (PDF 1260 kb) [file 12859_2016_1224_MOESM1_ESM.pdf]

Supplementary Materials for
"Incorporating topological information for predicting robust cancer subnetwork markers in human protein-protein interaction network"

Navadon Khunlertgit[1] and Byung-Jun Yoon[1,2,*]

[1] Department of Electrical and Computer Engineering, Texas A&M University, College Station, TX 77843-3128, USA
[2] College of Science and Engineering, Hamad bin Khalifa University (HBKU), P.O. Box 5825, Doha, Qatar
[*] Correspondence: Byung-Jun Yoon (bjyoon@ece.tamu.edu)

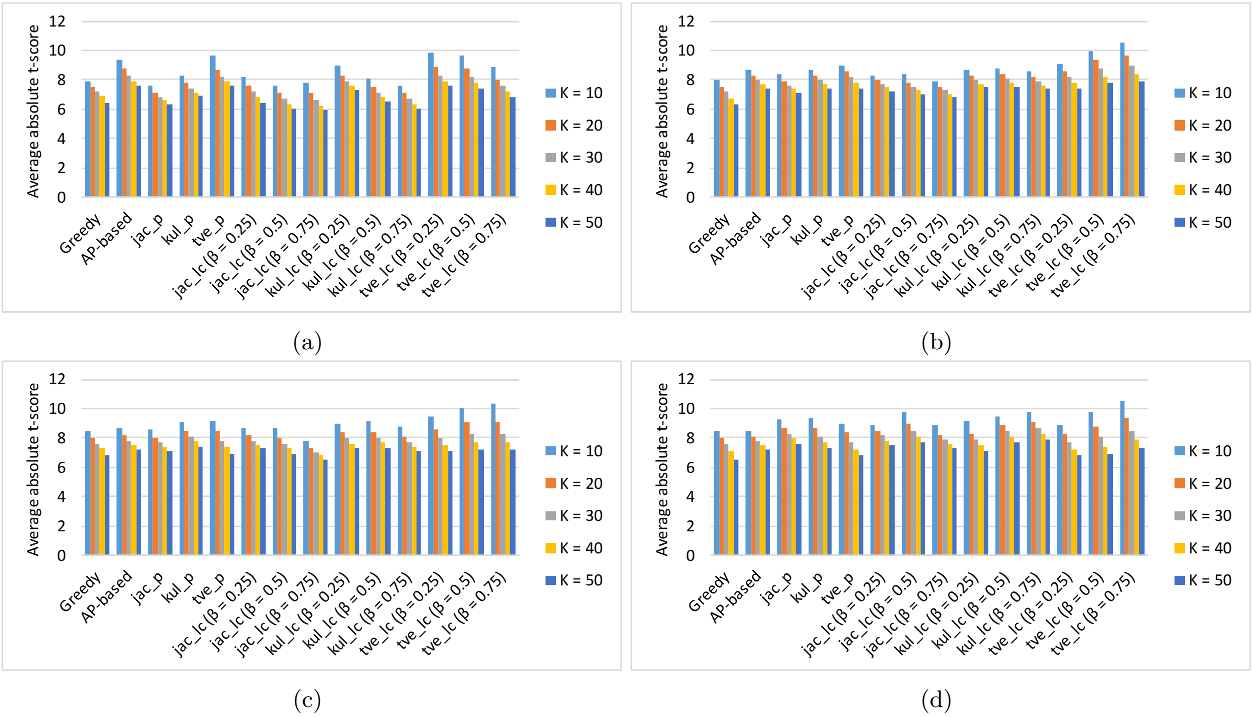

Figure S1: **Discriminative power of subnetwork markers identified on NKI295 by different methods.** We evalutated the discriminative power of the top K=10, 20, 30, 40, and 50 subnetwork markers identified on NKI295 by varied methods for the following PPI datasets: (a) Chuang, (b) HPRD, (c) GASOLINE, and (d) BioGRID.

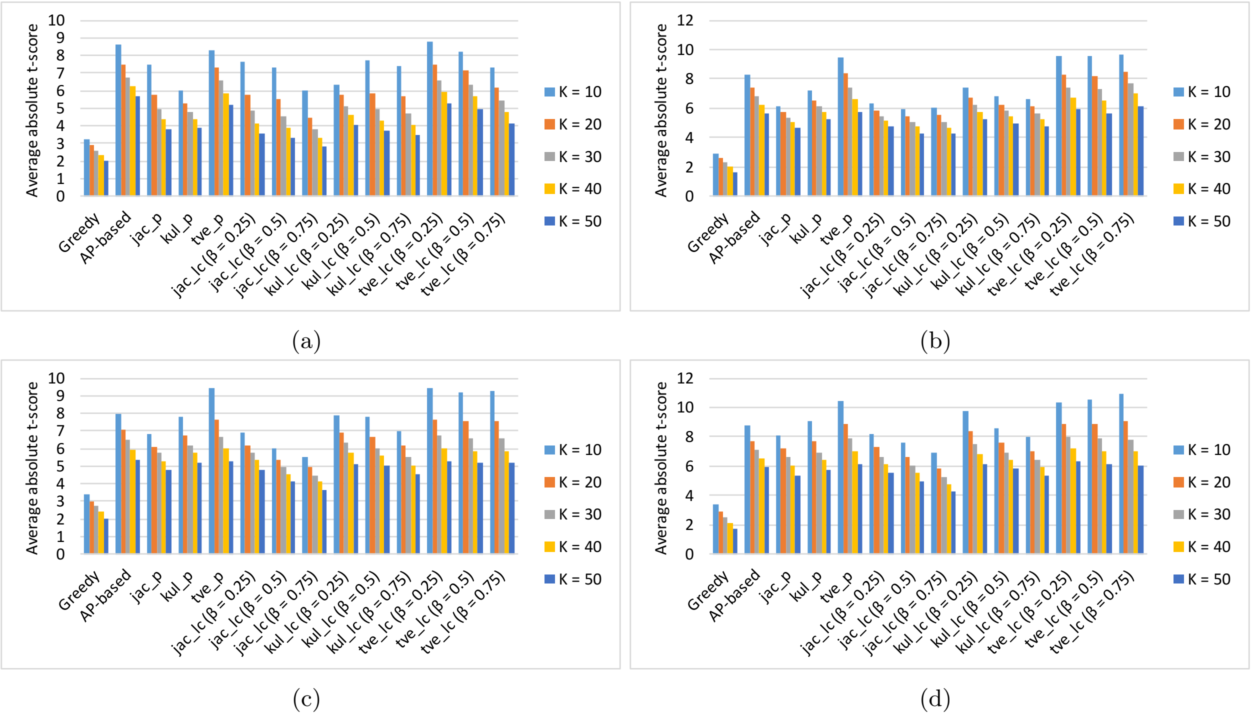

Figure S2: **Discriminative power of subnetwork markers across independent gene expression datasets.** The markers were identified and sorted on NKI295 and their discriminative power was evaluated on GSE2034. We repeated the cross-dataset discriminative power assessment for the following PPI datasets: (a) Chuang, (b) HPRD, (c) GASOLINE, and (d) BioGRID.
